# Deforestation for oil palm increases microclimate suitability for the development of the disease vector *Aedes albopictus*

**DOI:** 10.1038/s41598-023-35452-6

**Published:** 2023-06-12

**Authors:** E. S. Saager, T. Iwamura, T. Jucker, K. A. Murray

**Affiliations:** 1 Centre for Translational Immunology, University Medical Centre Utrecht, Utrecht, The Netherlands; 2 Department F.-A. Forel for Aquatic and Environmental Sciences, University of Geneva, Geneva, Switzerland; 3 School of Biological Sciences, University of Bristol, Bristol, UK; 4 MRC Unit The Gambia at London School of Hygiene and Tropical Medicine, Fajara, The Gambia; 5 MRC Centre for Global Infectious Disease Analysis, Imperial College London, London, UK

**Keywords:** Conservation biology, Ecological modelling, Ecological epidemiology

## Abstract

A major trade-off of land-use change is the potential for increased risk of infectious diseases, a.o. through impacting disease vector life-cycles. Evaluating the public health implications of land-use conversions requires spatially detailed modelling linking land-use to vector ecology. Here, we estimate the impact of deforestation for oil palm cultivation on the number of life-cycle completions of *Aedes albopictus* via its impact on local microclimates. We apply a recently developed mechanistic phenology model to a fine-scaled (50-m resolution) microclimate dataset that includes daily temperature, rainfall and evaporation. Results of this combined model indicate that the conversion from lowland rainforest to plantations increases suitability for *A. albopictus* development by 10.8%, moderated to 4.7% with oil palm growth to maturity. Deforestation followed by typical plantation planting-maturation-clearance-replanting cycles is predicted to create pulses of high development suitability. Our results highlight the need to explore sustainable land-use scenarios that resolve conflicts between agricultural and human health objectives.

## Introduction

Agricultural development has an extensive impact on natural and socioeconomic systems worldwide^1,2^. The globally rising demand in versatile tropical crops, such as oil palm, has led to a rapid increase in agricultural exploitation in developing countries^3,4^. Although agricultural development can bring important economic benefits, it has well known implications for biodiversity and carbon storage especially in areas where agricultural land directly replaces pristine tropical rainforest^5^. Currently, however, the measurable health risks of different land-use types remain poorly evaluated and human health impacts are therefore rarely integrated into land-use decision making.

Infectious disease transmission is an important dimension of human health that can be affected by agricultural land-use change. Patz et al*.*^6^ proposed that changing landscapes could become ‘unhealthy landscapes’ because of numerous examples of land-use change being linked to infectious disease risks (e.g., Lyme disease, Nipah virus). Since then, numerous studies have linked agricultural land-use change to increased infectious disease incidence^7–10^. In a recent meta-analysis, Shah et al*.*^11^ found that exposure to agriculture on average almost doubled the risk of being infected by any pathogen, with the highest effect sizes being observed for tropical tree crop monocultures including oil palm (odds ratio (OR) = 3.25) and rubber (OR = 2.27).

One mechanism that could help explain such associations is a change in microclimatic conditions following the change in land cover, which may favour the development of disease vectors^12,13^. Ectothermic arthropod vectors are highly sensitive to changes in environmental temperature, which govern their metabolic rates and development, and therefore their fitness and population growth rates^14,15^. In addition, many disease vectors respond to changes in humidity and/or rainfall due to their aquatic life-stages, which require the availability of adequate water bodies for development^15,16^. The precise effects of climate on population abundance are nevertheless highly vector-specific.

The changes in microclimate associated with deforestation for agricultural expansion offer considerable potential to impact disease vector development. Forest canopies typically buffer against extremes in local temperature and humidity through interception, transformation and storage of solar radiation, leaf transpiration and altered airflow^17^. Landscapes in transition to tropical tree-based agriculture, such as rubber or oil palm plantations, often encompass a strong gradient in land-use intensity with many differences in vegetation cover, canopy height and community complexity. Both on a relatively small spatial and temporal scale, vegetation in these landscapes can range from 50 + meter high intact or selectively logged tropical rainforest, to clear-cut open land, to 10–20 m full-grown plantation trees, all with varying microclimatic features. Recently, Jucker et al*.*^18^ performed high-resolution modelling of microclimate in a transitioning oil palm landscape and showed that deforested areas and oil palm plantations experienced substantially higher daily temperatures and lower relative humidity compared with rainforest areas. Older plantations experienced lower temperatures and higher relative humidity than younger plantations, although they remained warmer and drier than the rainforest areas. Predicting how these microclimate differences might in turn impact mosquito development and thereby, potentially, vector-borne disease (VBD) risks, is essential to be able to direct both large- (i.e. deforestation) and small-scale (i.e. cultivation design) land-use policy decision making.

Multiple field and experimental studies have demonstrated that land-use change can accelerate disease vector development through altering microclimates^19–22^. Mechanistic models of vector development could be an important tool to translate these observational findings into predictions across different settings, and, eventually, into land-use policies^23^. By explicitly incorporating environment-development relationships, they are also useful in evaluating the complex interplay of different environmental variables, such as temperature and humidity. Multiple studies have, for instance, already assessed the complex effects of global climate change on local disease vector development using mechanistic models and have predicted an acceleration of disease vector development resulting in expanding as well as shifting global distributions in the coming decades^24–28^. Mechanistic modelling studies that incorporate the effects of land-use change are scarcer and are typically limited by lack of adequately fine-scaled datasets that can capture the microclimatic variation attributable to vegetation cover differences^29,30^.

Here, we extended a recently developed spatially explicit physiological development model for the mosquito *Aedes aegypti*^28^ to predict the impact of fine-scaled, tree cover-related changes in microclimate on the number of life cycle completions of the related arboviral disease vector *Aedes albopictus*. Detailed land-use and microclimate data from Malaysia^18^ was used to capture the impacts of tropical forest conversion to oil palm plantation on mosquito development at appropriate spatio-temporal scales^31^. A comprehensive representation of the interactions between microclimates and vector development is provided by evaluating the separate and combined effects of temperature and humidity. We use our results to infer the trajectories of mosquito population growth according to realistic land-use succession scenarios within an oil palm-agricultural landscape. With this study, we aim to determine to what extent a forest-to-plantation transition could enhance mosquito development as a result of changes in local temperature and humidity linked to land-use change. Our results have important implications for evaluating vector-borne disease risks of agricultural expansion.

## Results

### Land-use change drives *A. albopictus* development suitability in low-land areas

**Figure 1:**
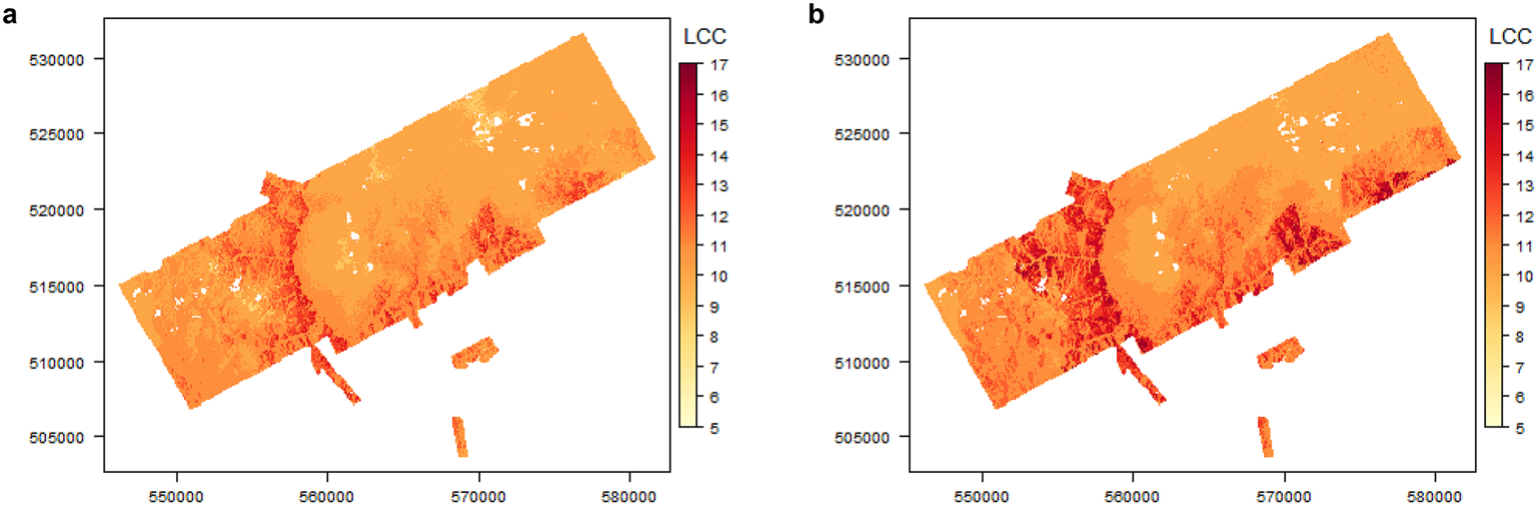
Map of the study site in the SAFE project area in Malaysian Borneo depicting the modelled total life-cycle completions (LCC/year, 2014) of A. albopictus. Figure shows clear spatial heterogeneity in vector suitability across a landscape in transition from rainforest to oil palm plantation for (**a**) combined (ER) model variation, predicting mosquito development based on temperature, evaporation and rainfall. (**b**) base model variation, including only temperature-based parameters for development.

Our full model (ER), that combines temperature, evaporation and rainfall, predicts substantial spatial heterogeneity in the number of successful life-cycle completions (LCC/year) for *A. albopictus* in the study region (Fig. 1). Across the study area, model output ranges between 5 and 15 LCC/year, with a mean suitability of 10.6 LCC/year. The suitability pattern is congruent with the distribution of the two main determinants of microclimate: land-use and elevation (Supplementary Figure S1).

**Figure 2:**
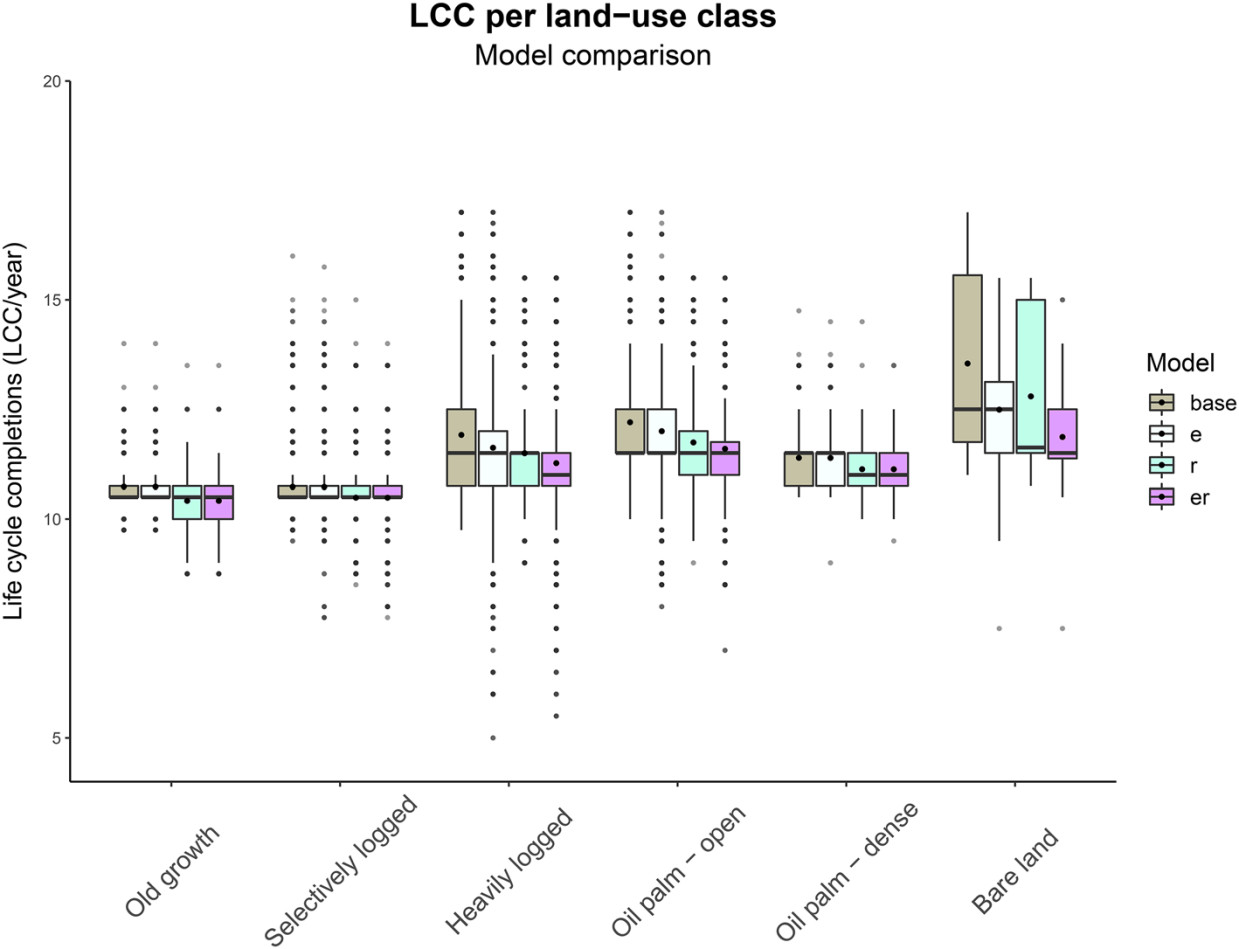
Comparison of total life-cycle completions (LCC/year, 2014) of A. albopictus between land-use classes and model variations. Figure shows highest vector suitability for the base model variation in the most modified land-use types. Model variations predict mosquito development with: Base = temperature, E = temperature + vapour pressure deficit (VPD) and rainfall for breeding-site evaporation, R = temperature + rainfall for egg-hatching, ER = combining E + R . Boxplot with bold black dots for mean LCC/year and normal dots for outlying values.

**Figure 3:**
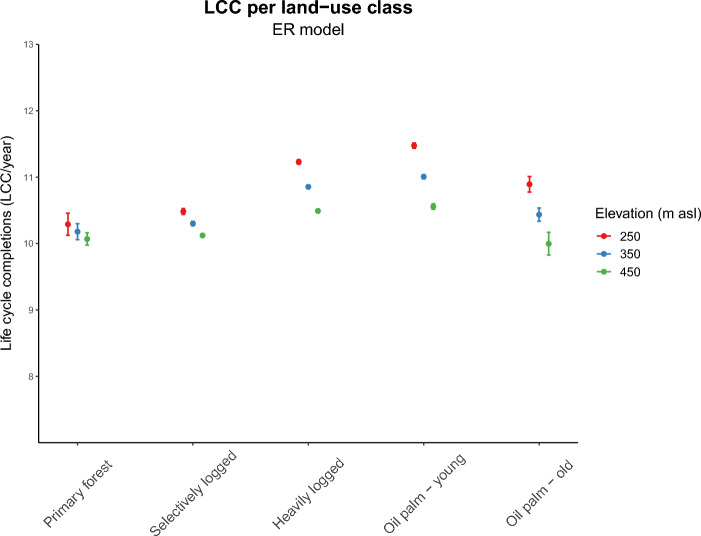
Predicted total life-cycle completions (LCC/year, 2014) of A. albopictus per land-use class at different elevation levels. Figure shows increased vector suitability during deforestation for oil palm plantation, with the strongest effect in low-land areas (250 m asl). Marginal effects analysis for the combined (ER) model output, with mean and 95% CI of LCC/year for three elevation levels and fixed aspect (0 radians).

Stratification of LCC/year by land-use indicates that vegetation cover loss following deforestation and oil palm conversion results in an increased suitability for *A. albopictus* development (Fig. 2; ER model). However, land-use and topography are strongly interrelated, as lowland areas are generally more beneficial for oil palm cultivation than highland areas. Marginal effects analysis, used to disentangle the effects of land-use versus topography, demonstrates that even after correcting for topography, vegetation cover loss remains a substantial driving force of increased development intensity of *A. albopictus* (Fig. 3)*.*

The ER model combined with a marginal effects analysis for topography and land-use predicts that development intensity increases by 10.8% in lowland areas when transitioning from primary forest (PF) to a young oil palm plantation (oil palm—young; OY) (absolute difference OY-PF: 1.1 LCC/year; Fig. 3; Supplementary Table S1). When the oil palms mature towards a higher and denser vegetation cover, environmental suitability decreases. However, also in older plantations (oil palm—old; OO) suitability remains increased compared to tropical forest areas (PF-OO: 4.7%). Young oil palm plantations and heavily logged forest differ minimally, as do primary and selectively logged forest. In all land-use classes, environmental suitability for *A. albopictus* decreases with elevation. However, the elevation effect is much stronger for converted land than for the forest areas, resulting in a decreased difference between young oil palm plantations and primary forest with elevation (OY-PF 250 m: 10.8%, 350 m: 7.9%, 450 m: 5.1%). At the upper elevation level, older oil palm plantations are no longer significantly different in vector development suitability compared to primary forest (OO-PF 450 m: 0.01%, n.s.). When the interaction terms between topography and land-use are removed from the generalized linear model, the difference between primary forest and young oil palm plantations is the same at all levels of elevation, namely 6.0% (ER model; supplementary Figure S2d).

The importance of the high resolution of our microclimate and land-use data is stressed by the results of model-runs on aggregated datasets. We show that already when aggregating our dataset to 500-m resolution, the significant difference between young and older oil palm plantations is lost at lower elevation (Supplementary Figure S2e).

### Model incorporation of rainfall and evaporation moderates deforestation-driven development intensity

In addition to studying full model results (ER model), we also modelled the separate impact of temperature versus humidity on vector development intensity following land-use change. In deforested land-use types we generally find higher temperatures, but lower humidity (Supplementary Figure S3). We show that the effect of decreased vegetation cover is most pronounced in the base model with only temperature (see also Fig. 1b, Fig. 2, Supplementary Figure S2a-). Increasing model complexity through the addition of both a rainfall/evaporation balance and a rainfall-threshold (ER model) negatively affects LCC/year in young oil palm plantations and heavily logged areas, whereas the model outcome is little affected in forest areas and older oil palm plantation. As a result, marginal effects analysis reveals a smaller average difference in environmental suitability between high and low vegetation cover areas for the ER model than for the base model (OY-PF base model: 12.4% vs ER model: 10.8%; HL-PF base model: 11.8% vs ER model: 8.2%; Supplementary Table S1). The effect of elevation is stronger for the ER model than for the base model (OY-PF base model 250 m: 12.4%, 350: 11.1%, 450 m: 9.7%).

When we compare the individual model variations (ER model, E model and R model, Supplementary Figs. 2b-d, Supplementary Figs. 6a-d), we observe that at lower elevation most of the effect in the ER model is mediated by the rainfall-triggered hatching threshold (R model: OY-PF 11.5%). As can be appreciated in Supplementary Figures S3a-c, primary forest indeed receives on average more rainfall than oil palm plantations, which could be related to cultivation preferences or deforestation constraints. The addition of the rainfall/evaporation balance for breeding sites mainly creates a larger spread in vector development suitability across the study site, especially towards lower suitability (Fig. 2; LCC_min_–LCC_max_ E model: 5–17, R model: 8–15, base model: 9–17). At higher elevation, the average difference between primary forest and young oil palm plantations is decreased for both the E and R model variation (OY-PF E model 250 m: 12.4%, 350: 9.7%, 450 m: 6.9%; R model: 11.5%, 9.2%, 7.0%).

We find that a subset of heavily logged cells exhibits a disproportionate decrease in LCC/year with the introduction of the rainfall/evaporation balance into the model (Supplementary Figure S2f.-g). Most of these cells were originally classified separately as ‘Bare land’, a category that has the lowest vegetation cover of all classes (mean canopy height: 2 m; Table 1) but that we incorporated into the ‘Heavily logged’ class due to the small size (28 cells; < 3 cells after spatial lagging). When we calculate marginal effects on the entire dataset without spatial correction, environmental suitability is highest of all classes in ‘Bare land’ in the base model, but shows the largest decrease after addition of the evaporation/rainfall balance in the E model, especially at higher elevation (BL-PF Base model 250 m: 28.5%, 450 m: 24.1% ; E model 250 m: 20.2%, 450 m: 6.6%; Supplementary Figs. 2e-f and Supplementary Table S1), illustrating the limiting effects of evaporation following vegetation-cover loss, despite the promoting effects of temperature.

**Table 1:** Land-use categories across the SAFE landscape.

Study ID	Land-use type	Mean Canopy height (m)	Grid cell count	% of total grid cells
1	Primary forest (PF)	58	4085	2.8
2	Selectively logged forest (SL)	31	53,304	36.7
3	Heavily logged forest (HL)	12	48,202	33.3
4	Oil palm plantation—young (OY)	8	35,683	24.6
5	Oil palm plantation—old (OO)	15	3755	2.6
6	Bare land (BL)	2	28	0.02

The table mentions the number and percentage of grid cells encountered within each class across the study site.

We explored the effects of varying model parameters according to the uncertainty in literature-derived estimates in a set of sensitivity analyses for the ER model (Supplementary Table S1). Although the magnitude of the difference in development intensity between land-use classes is somewhat variable depending on parameter settings (absolute difference OY-PF: 0.9–1.3 LCC/year), the overall trend is conserved significantly in all sensitivity analyses but wind speed. Increasing the average wind speed parameter by 1 mph reverses our results in the combined and E model, with lower suitability in open oil palm plantations and heavily logged areas compared to areas with higher forest cover (Supplementary Figure S2h).

### Land-use transition scenarios

**Figure 4:**
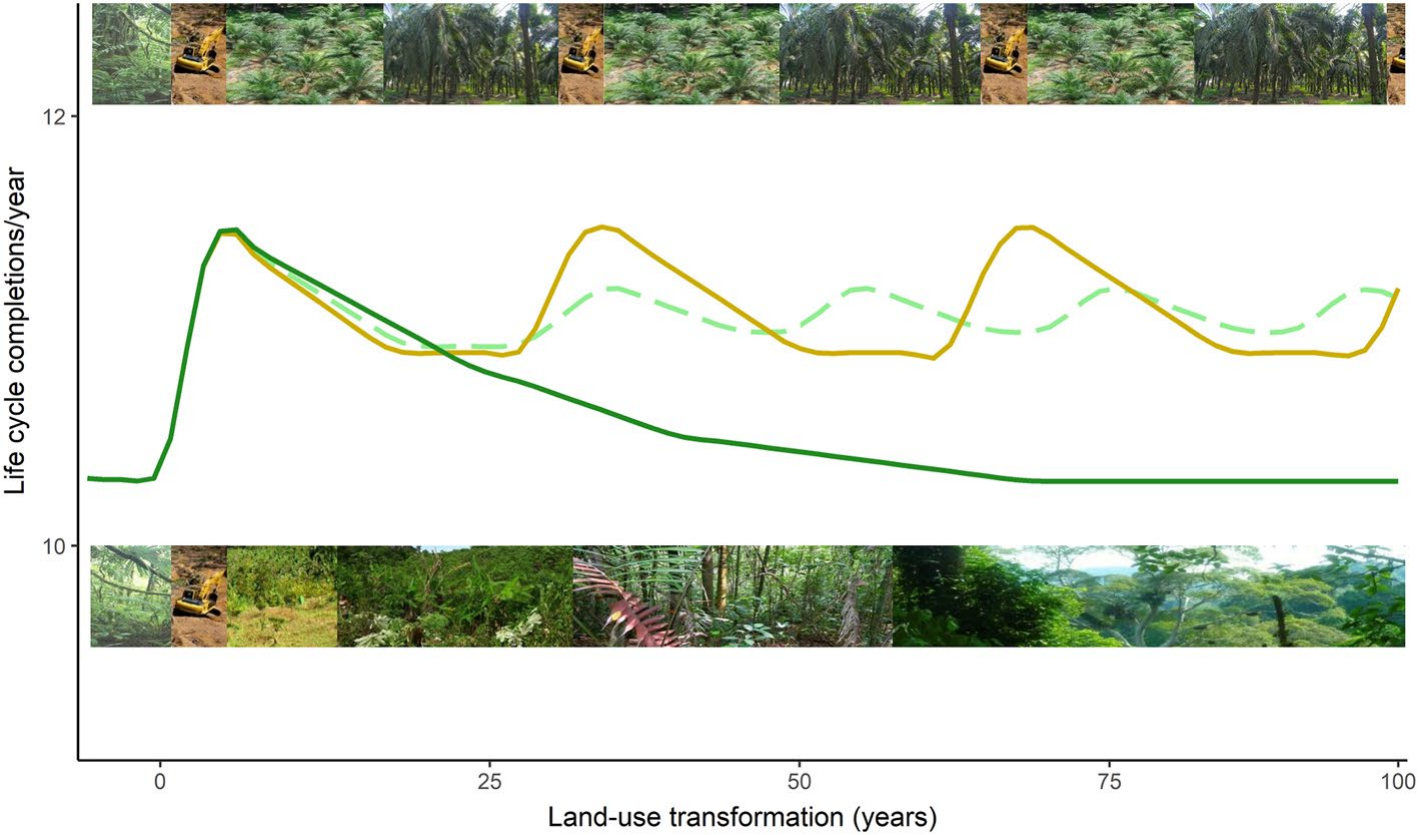
Potential development suitability (LCC/year) of Aedes albopictus along a 100-year time course exploring three hypothetical land-use transformation scenarios following deforestation. Dark green: Forest regeneration leading to gradually decreasing vector suitability. Yellow: Conventional oil palm plantation experiencing fluctuating, increased vector suitability during planting-maturation-clearance-replanting cycles. Light green: Alternative cultivation system experiencing more stable vector suitability due to e.g. co-cultivation of palms of different ages.

Our results predict a considerable impact of deforestation and oil palm conversion on the environmental suitability for *A. albopictus* through land-use related microclimate changes. In Fig. 4, we visualize how the results of the full model (ER model) would translate to a 100-year timeframe of oil palm development. Following the surge in disease vector suitability after the initial deforestation event, suitability is moderated as the oil palms grow in height and density. Since oil palm is a fast-growing crop, suitability is initially more moderated in plantations than when the land is left to regenerate back to natural tropical forest vegetation. However, as oil palm is commonly cultivated in 25-year planting-clearance-replanting cycles, the plantation landscape would be characterized by pulses of increased suitability after plantation clearance/replanting, followed by only transitory timeframes of gradually moderated suitability during plantation maturation. We envision that these peaks in suitability could be moderated by more sustainable agriculture systems that co-cultivate palms of different ages and/or integrate oil palms with preserved rainforest sections on a small scale.

## Discussion

An increased burden of vector-borne disease (VBD) is being highlighted as a major health risk of agricultural land-use change^6,11^. Assessing land-use related public health risks is therefore essential to design land-use policy that promotes sustainable socio-economic development. However, in contrast to global climate change, fine-scaled data of land-use related microclimate change have rarely been coupled to models of disease vector development. How the effects of small-scale changes in temperature and humidity will balance out with respect to vector development in a complex, tropical land-use transition landscape remains understudied. Here, we deployed a phenology model to study *Aedes albopictus* development intensity following deforestation and during the transition to oil palm plantation at the SAFE project site in Malaysian Borneo. We demonstrate that land-use driven microclimate change creates substantial differences in the number of total life-cycle completions (LCC/year) the mosquito can achieve across the study site (Fig. 3). Our combined (ER) model of temperature, evaporation and rainfall predicts that young oil palm plantations in lowland areas experience 10.8% accelerated development of *A. albopictus* compared to forest sites. Oil palm growth to maturity moderates suitability, but older plantations remain 4.7% more favourable for mosquito development than original tropical forest areas. This predicted effect size compares to the results of previous modelling studies of *Aedes Aegypti* abundance^26^ and LCC/year^28^ under global climate change. Hereby, we demonstrate that deforestation for agriculture—occurring in a time-span of months—could increase disease vector development intensity to an extent that might be comparable to decades of global climate change.

The transitional aspect of the landscape in addition to the cyclic nature of current oil palm cultivation practices adds an important temporal element to the difference in vector development suitability across our study site. Translating our spatial results into a century of hypothetical oil palm transition landscapes illustrates that the benefit of declined development intensity in mature plantations is transitory (Fig. 4). In the current cultivation practice of 25-yearly clearance-replanting-maturation cycles, oil palm plantations will likely experience recurrent pulses of increased temperature, increased development intensity and thereby potentially increased VBD risk. The impact of a land-use change event (i.e. deforestation) on VBD risk has been predicted before to change over time due to changing socioeconomic population dynamics^29^. Similarly, urban landscape design strategies were shown to have divergent effects on the local microclimate over time^32^. With a focus on microclimate-vegetation cover dynamics, our study now further emphasizes that VBD risk is not static in relation to a deforestation event, but is still highly influenced and thus manageable by subsequent land-use decision making. We suggest that including human-vector dynamics could further magnify these cyclic changes in VBD risk, as recently planted palm trees likely require more labour activity, which would increase the chances of human-vector contacts.

Our base model’s performance was successfully validated for *A. aegypti* by Iwamura et al*.*^28^ at a global scale, who showed a positive correlation between LCC/year and mosquito occurrence. However, we currently lack dense *A. albopictus* occurrence records of our study area to validate the predictions of our extended model for this species at this very local scale. In an extensive study that collected mosquitoes along a forest-plantation gradient, *A. albopictus* abundance—being the only species collected deep inside plantations—did not differ significantly, while mosquito species diversity and overall mosquito abundance decreased with distance into oil palm plantations^33^. A smaller study indicated increased *A. albopictus* abundance in agricultural land compared with forest, but unaltered overall mosquito abundance^34^. At the SAFE site in Borneo, Gregory et al*.*^35^ reported increased capture of *A. albopictus* on human landing sites in settlements located in oil palm plantations compared to those in logged forest areas. These studies, in conjunction with the results we present here, illustrate that land-use change can have a complex impact on disease vectors through both microclimate and a range ecological variables such as host availability^36^, interspecies competition^37^, larval and male nutrition^37,38^ and the number and types of breeding habitats^39,40^. A competitive larval advantage, especially in limiting conditions, is thought to be driving displacement of *A. aegypti*—the current main dengue vector—by *A. albopictus* in some but not all areas in North- and South-America^41^*,* which emphasizes the complexity of microclimate and ecological effects. Fine-scale studies that combine local occurrence records, microclimate and ecological measurements with mechanistic modelling are an important next step to comprehensively determine the impact of land-use change on mosquito development. This is of particular importance for infectious diseases such as dengue that are carried by multiple, competing vectors that differ in their anthroponotic and zoonotic transmission efficiency^41,42,42^.

Along with studying full model results, we analysed the separate impact of rainfall, evaporation, and temperature on *A. albopictus* development intensity. Whereas the increased temperature following deforestation is predicted to accelerate vector development, increased evaporation could start to limit breeding site availability in converted areas (Fig. 2). On average, this effect is modest and we predict high vector suitability in human-modified areas across all model variations. However, the models extended with breeding site evaporation (E and ER), do experience increased variation towards lower suitability, with some deforested cells supporting only 5 LCC/year in the E model. From literature, we know that *A. albopictus* abundance correlates not only with temperature, but also with rainfall and humidity^36,45,46^. In addition to providing a tool for better insight into the different microclimate and topographic factors that influence vector development, we therefore think that we increased the biological validity of our model by the rainfall and evaporation extensions in the ER model compared to the previously published base phenology model^28^. Validation with occurrence records would be an important next step to test the performance of the different model variations and improve prediction certainty.

In our study, we find that the predicted difference in suitability between land-use types is not only influenced by the model set-up, but also by a couple of data and variable parameters. First of all, we observe that vector development suitability is predicted to be more similar across land-use classes at higher elevation (Fig. 3), which is in line with earlier reports of increased microclimate buffering of forest canopies at higher temperatures^43,44^, as are found in low- versus highland areas. The differences are even further decreased when we include the evaporation/rainfall balance into our model, as VPD tends to increase with elevation across our study site (supplementary Figure S3c). Possibly, this could translate into a somewhat smaller public health risk of highland compared to lowland plantations. Although today oil palm is mainly cultivated below 300 m. asl because of higher yields^47,48^, our observation could have implications for future agricultural expansion, as highland cultivation is being explored in relation to rising temperatures under global warming^48^. Secondly, we find that we can no longer distinguish the difference in suitability between young and old plantations when we decrease the resolution of our dataset from 50 to 500 m. Previous modelling studies of mosquito development and VBD risk also emphasized the importance of high-resolution microclimate data, both in an urban context^49^ and in an agricultural context to delineate differences in VBD risk between crops^50^. Lastly, our sensitivity analyses indicate that the evaporation/rainfall balance might be substantially influenced by local wind speed differences. With increased wind exposure following vegetation clearance, evaporation of breeding sites is predicted to out-limit vector development in clear-cut land and open oil palm plantations compared to forest sites. However, we presumably overestimated the impact of wind on evaporation for a container breeding species as *A. albopictus* by using a simple evaporation/rainfall formula on a per-grid basis. The preferred natural and artificial breeding sites of *A. albopictus*, such as tree holes and used tires^51^, might each have a different, lower exposure to wind than open-water breeding sites. We now estimated a universal wind speed level for the entire study area, without accounting for the influence of vegetation cover on local wind speed. High resolution wind speed data and better knowledge of the relative impact of wind on breeding sites across different land-use types could help to better resolve the relationship between land-use, microclimate and vector development.

Our predicted increase in development suitability for the disease vector *A. albopictus* suggests a strong potential health co-benefit of preventing new deforestation events for palm oil development. Such co-benefits are highlighted as important goals to achieve more sustainable palm oil in the future^52^. Furthermore, the decrease in suitability predicted by our combined model when transitioning from young/open to old/dense plantations has important practical implications in the evaluation of alternative cultivation systems. Next to biodiversity benefits, previous research has shown that cultivation methods such as variable retention or mosaic landscaping can offer increased and more stable microclimate buffering compared to a conventional system^53,54^, which might help to prevent the predicted peaks in disease vector suitability during clearance-replanting phases^53,54^. The obtained microclimate buffering effect of these alternative cultivation systems would depend on the retained canopy cover, fragment size, distance between mosaic elements and the strength of the microclimate edge effect^55^. Future studies are necessary to determine how these factors would play out in an alternative oil palm cultivation system and could use our model in combination with other evaluation tools to determine a system that optimally balances fruit production with biodiversity conservation and infectious disease control.

## Conclusions

We predict that microclimate change by deforestation for oil palm, which can occur in a mere months, could increase disease vector development suitability to an extent comparable to decades of global warming. Our model predicts that plantation maturation to higher vegetation cover moderates *A. albopictus* development rates, although suitability remains increased in lowland plantations compared to original forest areas. These results stress the importance of tropical forest protection and give directions for alternative oil palm plantation systems that minimize public health risks^12,28^.

## Methods

### Study site

**Figure 5:**
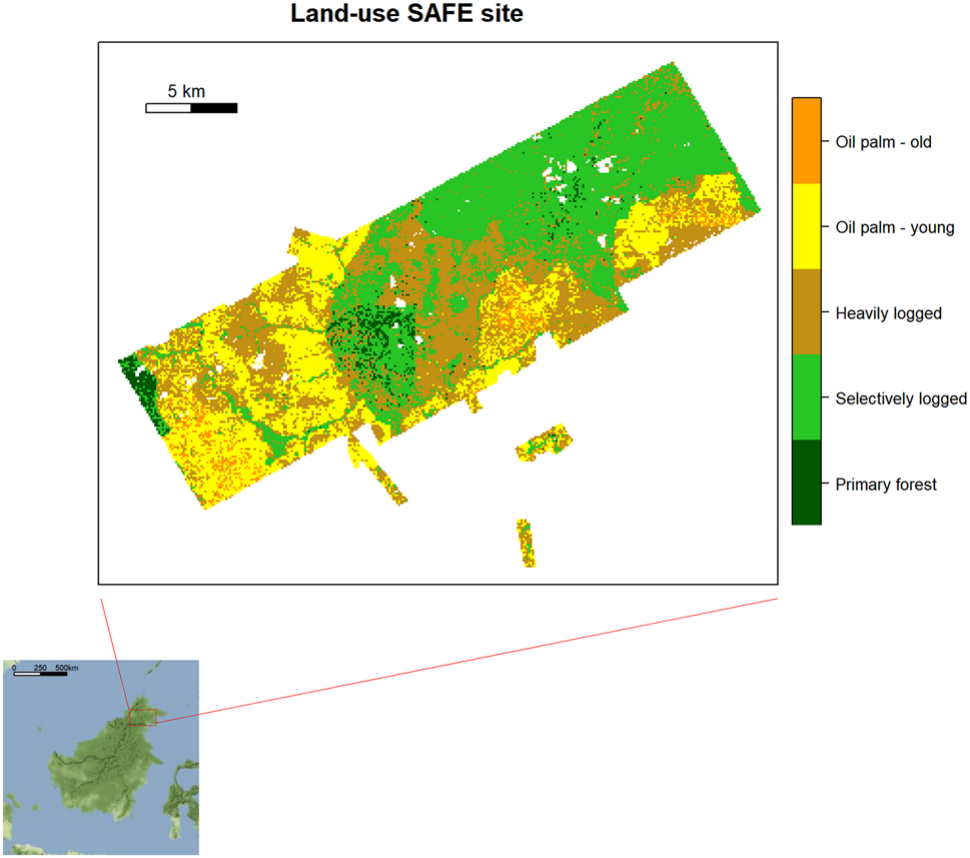
Land-use across the Stability of Altered Forest Ecosystems (SAFE) landscape classified at 50 × 50 m resolution based on different canopy metrics derived from Airborne Laser Scanning (ALS) data. The land-use categories include tropical rainforest in successive phases of disturbance and with decreasing canopy height and complexity (Primary forest, Selectively logged and Heavily logged), deforested areas recently planted with oil palms (Oil palm—young) as well as older plantations with increased canopy height (Oil palm—old). Due to its small size (28 cells), the land-use category ‘Bare land’ is excluded from the main analysis and is not visualized on this map. The inset shows the location of the SAFE landscape on the island of Borneo, South East Asia. Maps were made using raster and ggmap packages^59,89^.

The study area is located in Sabah, Malaysian Borneo (Fig. 5). The site is part of the Stability of Altered Ecosystems (SAFE) project, which is one of the largest ecological experiments in the world studying the impacts of deforestation and forest fragmentation on biodiversity and ecosystem functioning^57^.

The SAFE landscape is designated for planned conversion into oil palm plantation and sustains an extensive forest modification gradient. Experimental plots include unlogged primary forest (located in and adjacent to the Maliau Basin Conservation Area), selectively and extensively logged forest (including fragmented forest areas), and oil palm plantation sites that include both recently planted and mature oil palms. Originally, land-use within the SAFE landscape was classified into 9 classes based on canopy metrics derived from Airborne Laser Scanning (ALS) data^58^. We reclassified the land-use into 6 overarching classes (Table 1). The terrain, land- use, rainfall and microclimate characteristics of the study site are provided in supplementary Figures S1 and S3. For more details, see Jucker et al. (2018).

### Data

#### Microclimate: air temperature and vapour pressure deficit

Microclimate data consisting of daily mean and maximum air temperature and vapour pressure deficit (VPD) at 50-m resolution were generated for the 2014 calendar year following the approach described in Jucker et al. (2018). To achieve this, hourly measurements of near-surface air temperature (T, °C) and relative humidity (RH, %) were acquired at 113 locations across the SAFE landscape (including primary forests, moderately and heavily logged forests and oil palm plantations). RH and T were used to calculate VPD (hPa) using Eq. (1):

1 $${\text{VPD}} = \left( {\frac{{100 - {\text{RH}}}}{{{\text{RH}}}}} \right) \times {\text{ e}}_{{\text{s}}} {\text{with e}}_{{\text{s}}} = 6.112{ } \times {\text{e}}^{{\frac{{17.67 \times {\text{T}}}}{{{\text{T}} + 243.5}}}}$$

Microclimate data on T and VPD were then combined with topographic and canopy structural information derived from airborne laser scanning (ALS) data acquired at the SAFE site in November 2014. The ALS data were used to generate a 1-m resolution digital elevation model (DEM) and canopy height model (CHM) of the study area, from which the following metrics were calculated at 50-m resolution for each of the 113 locations where microclimate readings were taken: elevation, slope, aspect, topographic position index, maximum canopy height and plant area index. Multiple regression models were then fit to predict annual mean and maximum air temperature (T_mean_, T_max_) and VPD (VPD_mean_, VPD_max_) based on the topographic and canopy structural metrics across the entire SAFE landscape (approximately 363 km^2^) at 50-m resolution. Finally, to generate daily values of T_mean_, T_max_, VPD_mean_ and VPD_max_ for each 50-m grid cell of the landscape, we used the field data to calculate an offset value capturing the difference between mean annual values of T_mean_, T_max_, VPD_mean_ and VPD_max_ and those observed in each day of 2014 across all microclimate stations. This daily offset was then added to the mean annual values predicted for each 50-m grid cell, creating 365 gridded maps of T_mean_, T_max_, VPD_mean_ and VPD_max_ of the SAFE landscape. This approach assumes that the underlying relationship between microclimate, topography and canopy structure remains constant across seasons, which in the case of Sabah is likely a reasonable assumption given the region’s climate is relatively aseasonal.

#### Rainfall

Daily precipitation data were obtained from the Climate Hazards Group InfraRed Precipitation with Station data (CHIRPS) dataset, containing over 35 years of precipitation data (mm/day) between 50°S and 50°N at 0.05° resolution (= 5.55 km). We downloaded the global daily netCDF files for 2014 from "https://data.chc.ucsb.edu/products/CHIRPS-2.0/". Data were read into R using the *raster* package^59^ and were projected to fit the extent and resolution of the microclimate data.

#### Wind speed

One average wind speed value for the entire study area was estimated from the weather station in Sapulut, Malaysia, being the closest inland weather station to our study area. Data were obtained through "https://worldweatheronline.com/".

### Phenology model

We extended the mechanistic phenology model that was developed for *A. aegypti* by Iwamura et al. ^28^ to model the development of the closely related species *Aedes albopictus*. In short, the spatially explicit phenology modelling framework tracks development success of the female mosquito across four development stages: 1) egg hatching, 2) immature development (larvae + pupae), 3) blood feeding and 4) oviposition. Using thresholds and growing degree days (GDD) the model evaluates the gridded climate data with daily temporal resolution to determine progression to the next life stage (see overview supplementary Figure S4) and then calculates the number of successful life-cycle completions (LCC) per time step (here: 1 year). This output can be considered a development intensity index that theoretically relates to a species’ probability of occurrence and abundance in specific locations^28^.

In addition to adapting the model parameters to *A. albopictus* (see section *Model Parameters*), we modified the model as developed by Iwamura et al.^28^ in three ways:

First, we adapted the calculation of the adult life stages 3 and 4 to account for day-to-day variation in temperature by updating the time left until life-stage completion (*D*_*c*_) using Eq. (2):

2 $$D_{i}^{c} = D_{i - 1}^{c} - 1 - \frac{{(D_{max} - D_{t} )}}{{D_{t} }}$$

where the mean temperature at day *i* dictates *D*_*t*_* (*according to four different temperature ranges) with D_max_ fixed at the D_t_ of the lowest temperature range (= maximum duration of this life-stage). $D_{i}^{c}$ is then determined as the time left until life-stage completion at day *i*, with $D_{i}^{c}$ = *D*_*max*_ at the first day of the life-stage and $D_{i}^{c}$ being updated daily until $D_{i}^{c}$ = 0, after which the next life-stage is entered.

Second, we introduced a daily rainfall threshold to activate embryonated eggs into hatching. Although embryonation can progress above the water line^60,61^, hatching of the eggs requires inundation, supposedly triggered by low oxygen levels, microbial metabolites and/or plant chemicals in the water^62–65^.

Last, we expanded the model to incorporate the availability of suitable water bodies for the survival of larvae and pupae, which are very sensitive to desiccation. In the immature development stage, we set a threshold for the minimal water level that can support survival of a population of *Aedes albopictus* larvae and pupae, below which immature life-stages die and the model is reset to the egg stage. We fill each grid cell (2500 m^2^) with the daily volume of precipitation (P_grid_) using the CHIRPS data resolution (1000 ml/m^2^/day) using Eq. (3):

3 $$P_{grid} \left( {ml/day} \right) = P_{CHIRPS} *50*50*1000$$

then empty it by the daily volume of evaporation (*E*_*grid*_) using Eq. (4):

4 $$E_{grid} \left( {ml/day} \right) = \left( { - 0.18 + 0.125 \times VPD + 0.19 \times W} \right) \times 24 \times 1273560.9$$

with W wind speed in mph and VPD in mm Hg, following Kucera^66^, and then divide the net water volume by the grid cell surface (2500 m^2^) to calculate the potential breeding habitat depth (*H*) using Eq. (5):

5 $$H\left( m \right) = \frac{{(P_{grid} - E_{grid} )}}{50*50}$$

with death of immature development stages if *H* < *H*_min_.

The ability of *A. albopictus* to lay diapausing eggs with high cold and desiccation resistance allows the species to overwinter in colder regions^15,67,68^. However, as our study area experiences a tropical climate, lower temperature restrictions for egg survival were not included in our model.

We compared four model variations to evaluate the influence of precipitation, evaporation or both, in addition to temperature:

- Base model (Base): only temperature-based development
- Rain model (R): temperature-based development + daily precipitation threshold for egg hatching
- Evaporation model (E): temperature-based development + breeding site restrictions
- Full model (ER): combines the parameters of model E and R

#### Model parameters

From empirical literature we derived the following estimates for parametrization of environmental factors in *A. albopictus* development. The development times that we retrieved are specific for tropical strains of *A. albopictus* and often differ from temperate strains.

*Hatching* Best estimates for hatching parameters come from the review by Waldock et al*.*^15^, who derived an embryonation threshold of 15 °C.

For GDD, we fit a simple linear model through temperatures and development rates reviewed in Waldock et al*.*^15^ and took the inverse of the slope as an estimate of the GDD^69^. Points above 30 °C were excluded to ensure a linear relationship^70^. We derived a GDD of 57.8 (95% CI: 34.0–191.6). For sensitivity analyses we used the mean GDD ± 10 (47.8 and 67.8 GDD).

Most *A. albopictus* eggs are found around 2–10 mm above the water line^71^. We parametrized the rainfall threshold for hatching at 2 mm as we are interested in peak rather than mean distance. We conducted sensitivity analyses for rainfall levels of 0 and 10 mm as lower and upper values (R-low/R-high).

*Immature development* We set the lower threshold for immature development at 11°C^15,69,72^.

To estimate the GDD for immature development, we fit a simple linear model through the larval and pupal development times as reported across different studies^15,73–76^, excluding values above 30 °C. Since the results of Waldock et al*.*^15^ were aggregated from 5 reviewed studies, we weighted the values from Waldock 5 times more than those of the other 4 studies cited above. We derived a GDD of 180.5 (95% CI: 140.3–253.0). For sensitivity analyses, we used the mean GDD ± 10 (170.5 and 190.5 GDD).

Both for egg and immature development, increasing temperature stops to be beneficial above 30–35 °C ^15,69,74^. We conservatively parametrized a mean daily temperature of 30 °C as T_benefit_. As maximum T_mean_ in our dataset is 29.2 °C, this restriction was never applied in practice.

*Pre-blood stage* Based on Waldock et al*.*^15^, we estimated the maximum time (D_max_) between adult emergence and the first blood meal as 12 days, which decreases to 6 days if T_mean_ ≥ 20 °C, 4 days if T_mean_ ≥ 26 °C and back up to 10 days if T_mean_ ≥ 35 °C (D_t_).

*Gonotrophic cycle* Following Waldock et al*.*^15^ and Brady et al*.*^77^, we estimated the maximum duration (D_max_) of the gonotrophic cycle as 9 days, with 4 days if T_mean_ ≥ 26 °C, 3 days if T_mean_ ≥ 30 °C and 5 days if T_mean_ ≥ 35 °C (D_t_).

*Heat kill* Extreme temperatures can be a direct cause of mortality and are included as a heat kill threshold in our model. We parametrized T_heatkill_ at T_max_ ≥ 43°C^78,79^. As the highest T_max_ in our dataset is 40.6 °C, this restriction was never applied in practice. For sensitivity analyses, we tested a heat kill threshold of 40 °C.

*Breeding site availability* We set minimum water depth of breeding sites at 5 mm^80^ and performed sensitivity analyses for lower and upper water depth values (D-low/D-high) of 3.5 and 6.5 mm. We also performed sensitivity analyses on lower and upper values of the wind speed parameter in the evaporation rate formula (W-low/W-high): 1.2 and 3.0 mph, with mean wind speed 2.0 mph.

### Statistics

To isolate the impact of land-use change from the effects of topography*,* we used marginal effects analysis. We calculated the marginal effects on a generalized linear Quasi-Poisson model to account for the underdispersion of our count data (LCC/year), as indicated by dispersion testing (*AER* package^81^) and diagnostic residuals plots (*DHARMa* package^82^). The correlation matrix shows some correlation between elevation and slope, but assessment of the generalized variance inflation factors (*glmtoolbox* package^83^) did not indicate severe multicollinearity among our explanatory variables.

We compared several generalized linear Quasi-Poisson regression models for model selection with land-use and elevation as base explanatory variables, testing the additional explanatory value of aspect, slope and topographic position index (TPI), as well as interaction effects of topographic variables with land-use. To assess the performance of each of these models, we split our data into training (80%) and testing (20%) sets and compared training set-based predictions to the actual values of LCC/year in the testing set using R^2^, root mean squared error (RMSE) and mean absolute error (MAE). The following model (6) —including elevation, land-use, aspect and interaction effects—was selected for further analysis:

6 $$LCC = \, \beta 1 \, + \, B2*LU \, + \, \beta 3*E \, + \, \beta 4 \, *A \, + \, B6*E*LU \, + \, B7*A*LU$$

where β = regression coefficient; LU = Land-use, coded as a dummy-variable with Primary forest as the base variable; B = regression coefficient matrix for all dummy levels; E = elevation and A = aspect (sine transformed).

To assess the spatial autocorrelation in the residuals of our selected model we calculated Moran’s *I* (*spdep* package^84^) at different lag distances, using a row standardized weights matrix. Neighbours were determined as points lying within 50-m Euclidian distance (adjacent raster cells measured from centre). The correlogram depicted in Supplementary Figure S5a shows that there is considerable spatial dependency in our residuals. To minimize the impact of spatial autocorrelation on our analysis, we sampled cells from our output raster with a distance of 5 cells apart (Supplementary Figure S5b). We repeated 5-lag subsampling at different starting points and then averaged the model outcomes to represent the entirety of our data.

Using model (6) with spatially lagged model output, marginal effects were calculated to isolate the impact of land-use change on LCC (*ggeffects* package^85^). Mean LCC with 95% confidence interval (CI) was predicted for each of the 5 land-use classes (excluding ‘Bare land’ due to small class size) at 3 fixed elevation levels (250, 350 and 450 m asl) and 1 fixed level of aspect (mean aspect after sine-transformation: 0 radians). For the lower-resolution analysis, we ran the model on  a 500 m aggregated version of our microclimate dataset, using the mean function for the microclimate variables and, during analysis, the mean function for topographic and the modal function for land-use variables (*raster* package^59^).

We used the results from the marginal effects analyses to visualize *A. albopictus* development suitability over time across three different hypothetical land-use change scenarios. In the first scenario, deforested land is converted to oil palm plantations, which are cleared and replanted every 25–30 years when fruit production declines^53^, following common farming practice. In the second scenario, deforested land is abandoned after logging and is allowed to slowly regenerate back to a mature forest^86–88^. The third scenario envisions an alternative cultivation system, with e.g. co-cultivation of palms of different ages, as described in the text.

Analyses were performed in R version 4.1.2.

## Data and code availability

The microclimate data of daily mean and maximum temperature and VPD are publicly available at https://doi.org/10.5281/zenodo.7893600. Additional data and code are available upon request.

## Supplementary Information

Supplementary Information.
