# Supplementary material for: Deforestation for oil palm increases microclimate suitability for the development of the disease vector Aedes albopictus
Source: Sci Rep. 2023 Jun 12;13:9514. doi: 10.1038/s41598-023-35452-6 (PMC10260943; doi:10.1038/s41598-023-35452-6)
Supplement: Supplementary file 1 — Supplementary Information. [file 41598_2023_35452_MOESM1_ESM.pdf]

# Supplementary material

## Deforestation for oil palm increases microclimate suitability for the development of the disease vector *Aedes albopictus*

Saager, E.S.*[1], Iwamura, T.[2], Jucker, T.[3], Murray, K.A.[4]

1. Centre for Translational Immunology, University Medical Centre Utrecht, The Netherlands

2. Department F.-A. Forel for Aquatic and Environmental Sciences, University of Geneva, Geneva, Switzerland

3. School of Biological Sciences, University of Bristol, United Kingdom

4. MRC Centre for Global Infectious Disease Analysis, Imperial College London, United Kingdom, MRC Unit The Gambia at London School of Hygiene and Tropical Medicine, The Gambia

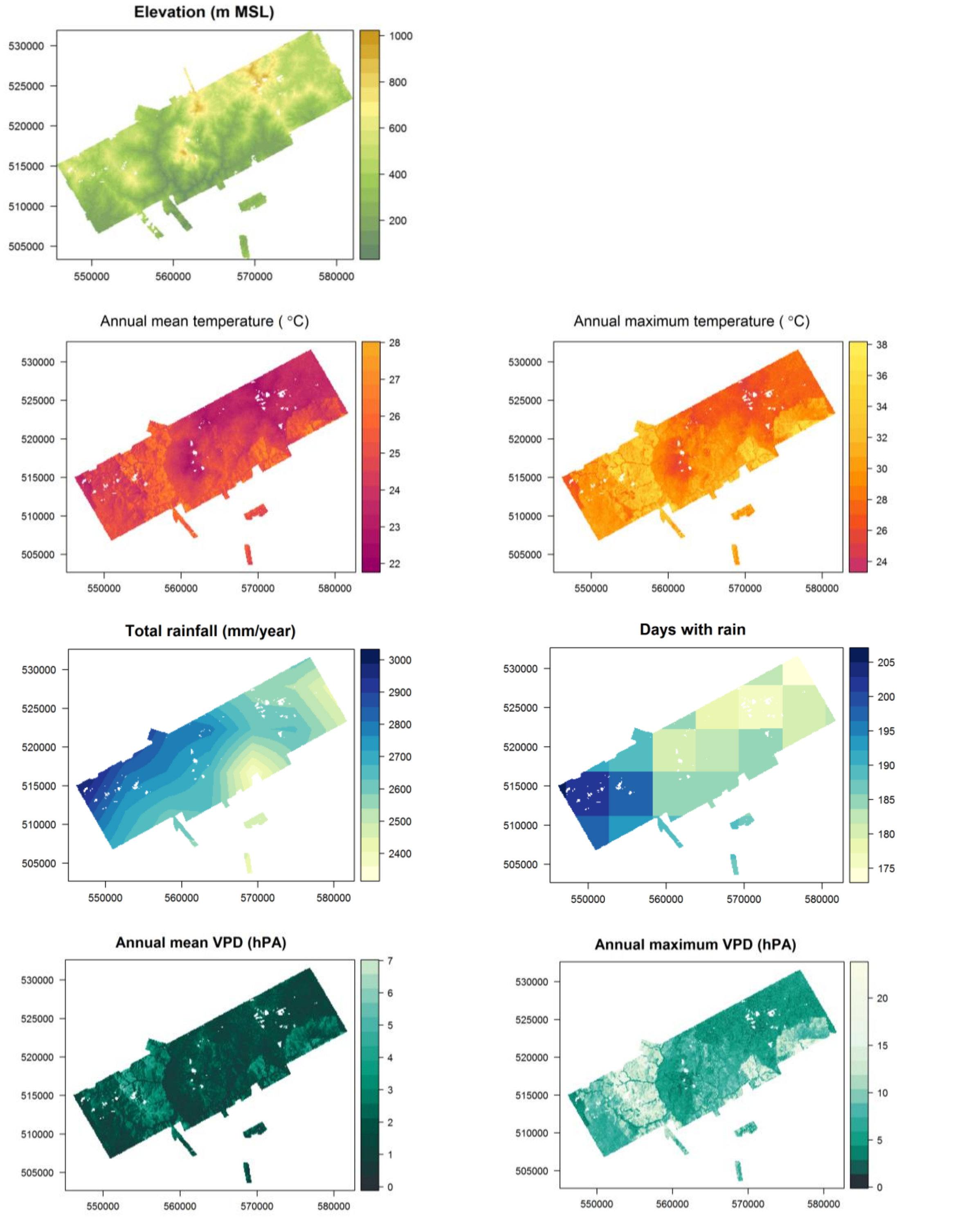


Supplementary Figure S1: *Distribution of important environmental and topographical variables in the study area.*

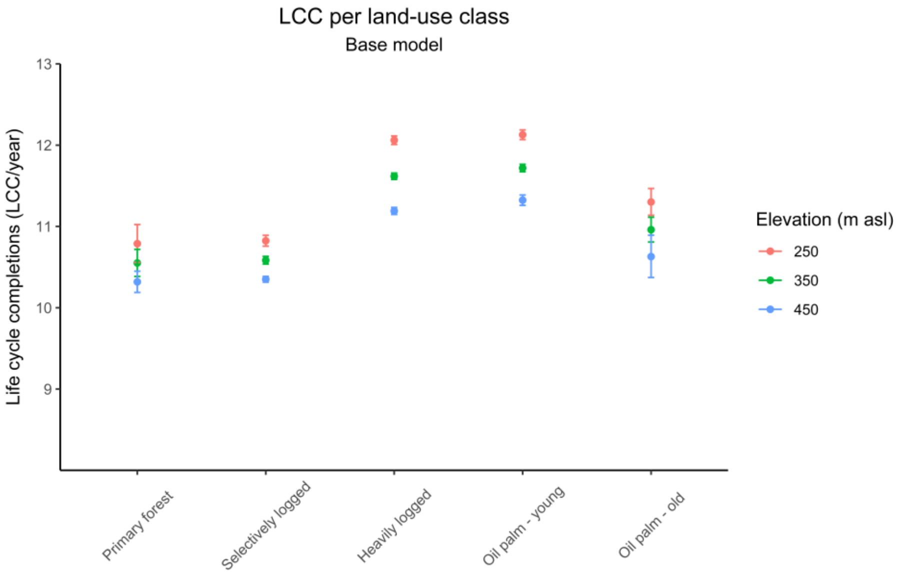
LCC per land-use class
Base model
Life cycle completions (LCC/year)
13
12
11
10
9
Primary forest
Selectively logged
Heavily logged
Oil palm - young
Oil palm - old
Elevation (m asl)
250
350
450

a)

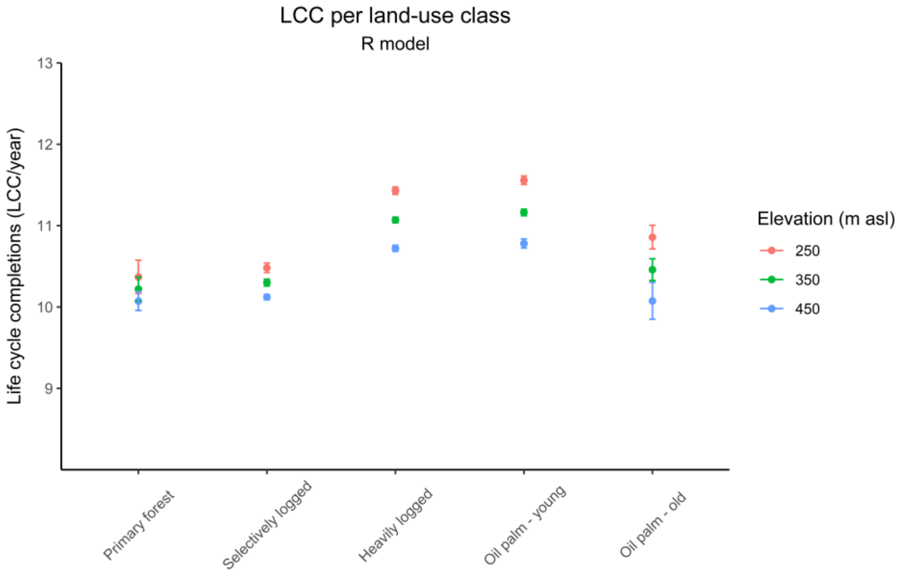
LCC per land-use class
R model
Life cycle completions (LCC/year)
13
12
11
10
9
Primary forest
Selectively logged
Heavily logged
Oil palm - young
Oil palm - old
Elevation (m asl)
250
350
450

b)

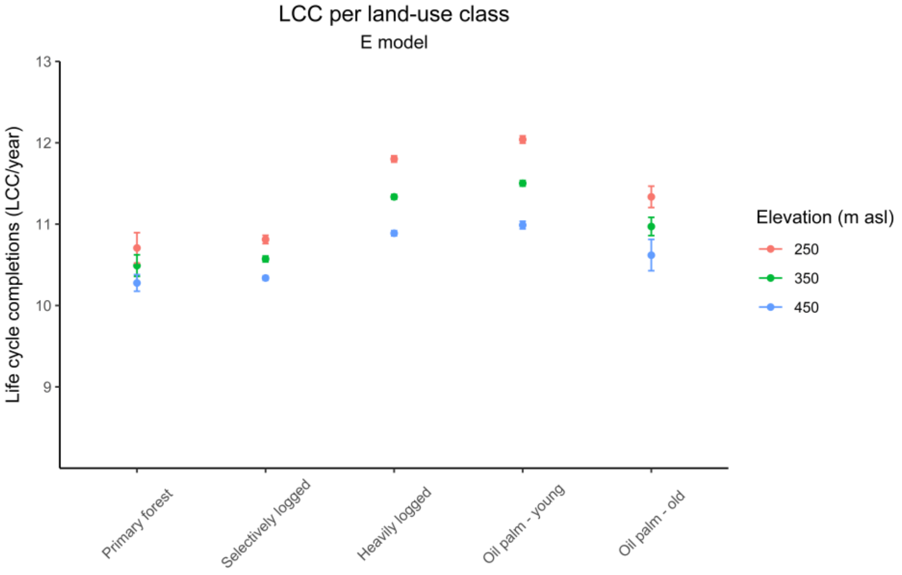
LCC per land-use class
E model
Life cycle completions (LCC/year)
13
12
11
10
9
Primary forest
Selectively logged
Heavily logged
Oil palm - young
Oil palm - old
Elevation (m asl)
250
350
450

c)

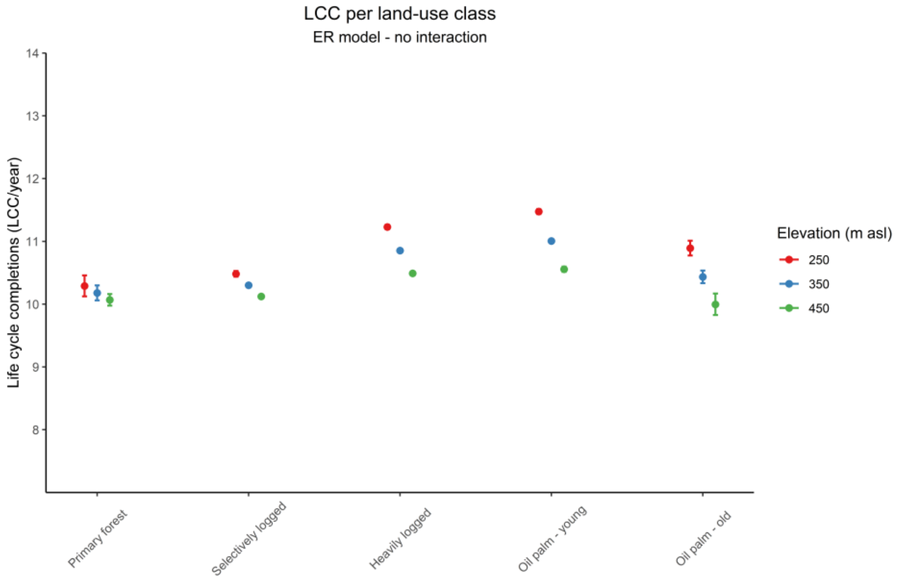
LCC per land-use class
ER model - no interaction
Life cycle completions (LCC/year)
14
13
12
11
10
9
8
Primary forest
Selectively logged
Heavily logged
Oil palm - young
Oil palm - old
Elevation (m asl)
250
350
450

d)

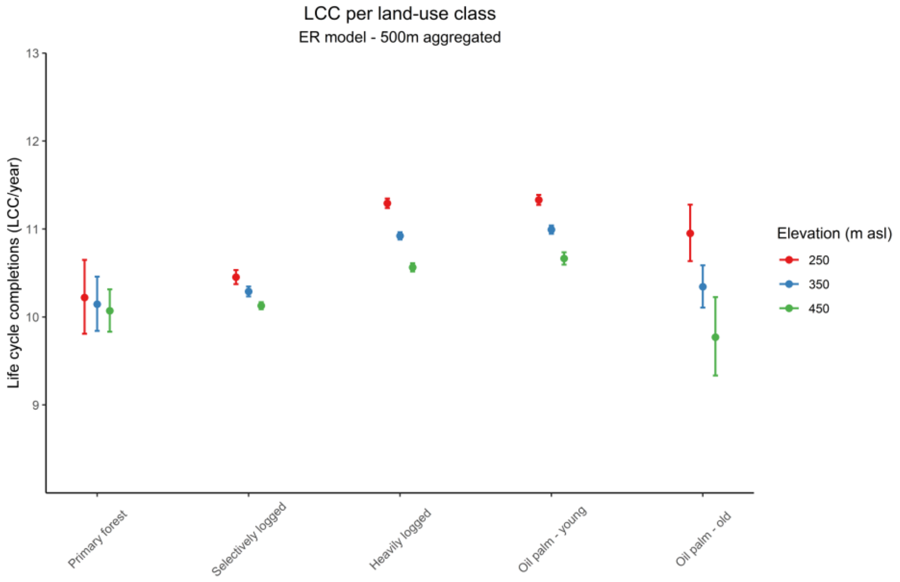
LCC per land-use class
ER model - 500m aggregated
Life cycle completions (LCC/year)
13
12
11
10
9
Primary forest
Selectively logged
Heavily logged
Oil palm - young
Oil palm - old
Elevation (m asl)
250
350
450

e)

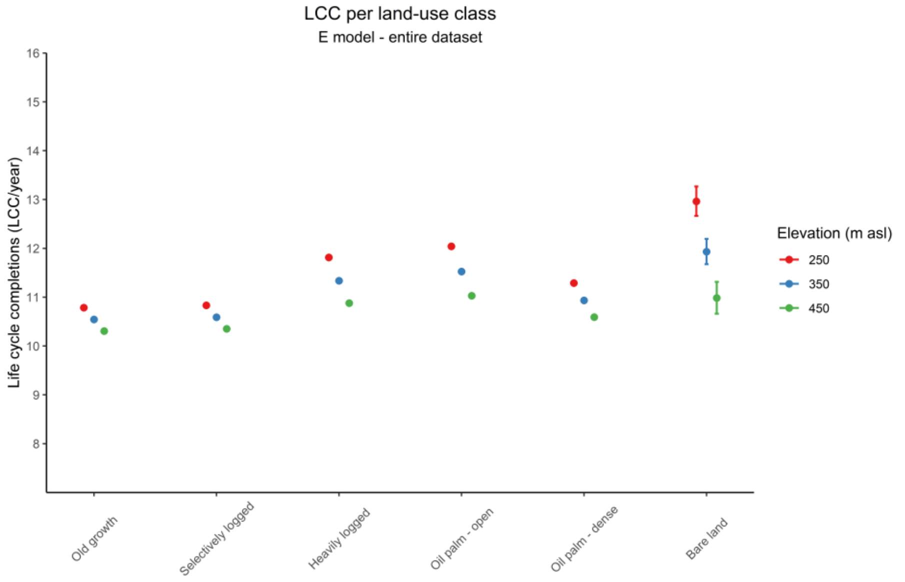
LCC per land-use class
E model - entire dataset
Life cycle completions (LCC/year)
Old growth
Selectively logged
Heavily logged
Oil palm - open
Oil palm - dense
Bare land
Elevation (m asl)
250
350
450

f)

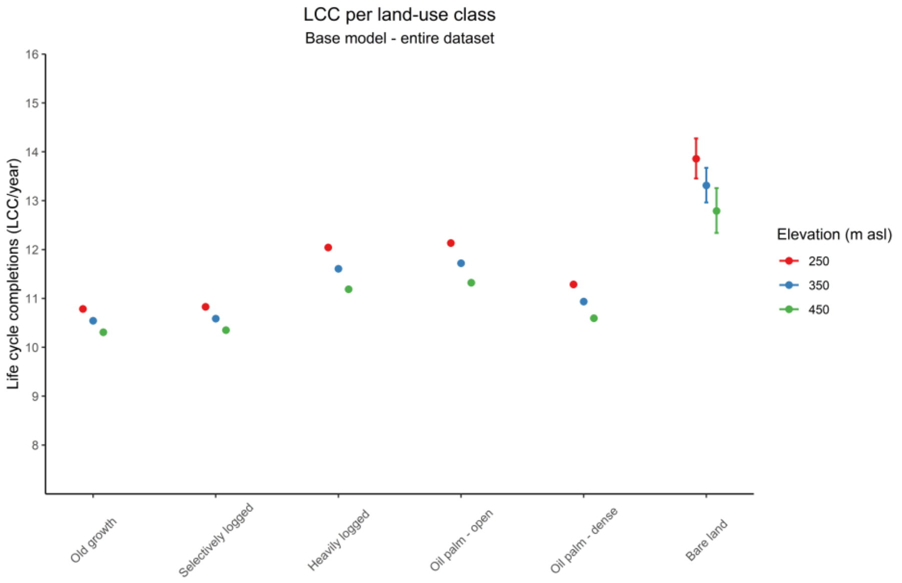
LCC per land-use class
Base model - entire dataset
Life cycle completions (LCC/year)
Old growth
Selectively logged
Heavily logged
Oil palm - open
Oil palm - dense
Bare land
Elevation (m asl)
250
350
450

g)

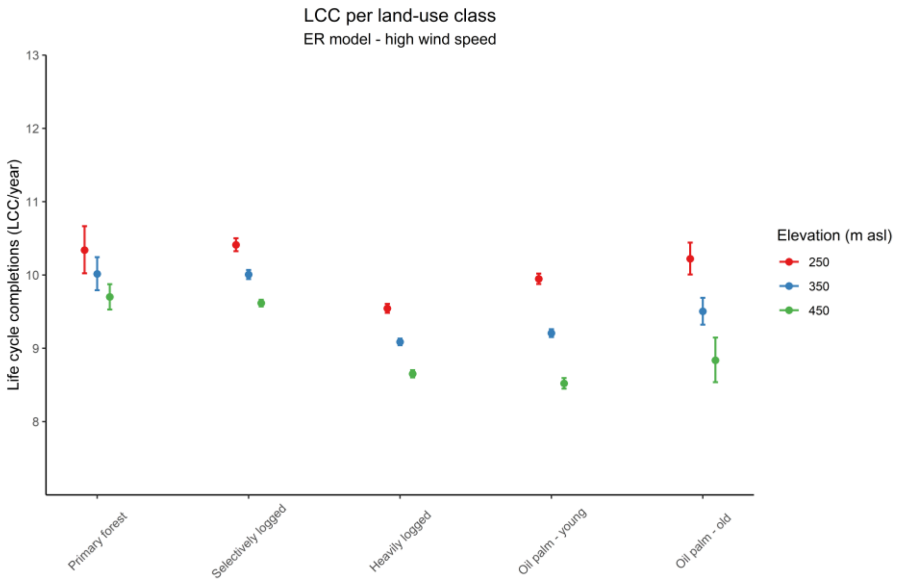
LCC per land-use class
ER model - high wind speed
Life cycle completions (LCC/year)
Primary forest
Selectively logged
Heavily logged
Oil palm - young
Oil palm - old
Elevation (m asl)
250
350
450

h)

Supplementary Figure S2: *Marginal effects analysis fixed at three different elevation levels (250,350,450 m asl) with sine-transformed aspect fixed at 0 radians, to determine the number of life-cycle completions (LCC) in the year 2014 across the different land-use types as encountered in the SAFE project area for model variations a) Base, including only temperature input b) R, including input of temperature and rainfall for egg-hatching c) E, including input of temperature and evaporation rates d) ER, no model interaction terms between topography and land-use e) ER, dataset aggregated to 500m resolution f) E, marginal effects analysed on the entire dataset g) Base, marginal effects analysed on the entire dataset h) ER, high wind speed (3 mph) sensitivity analysis*

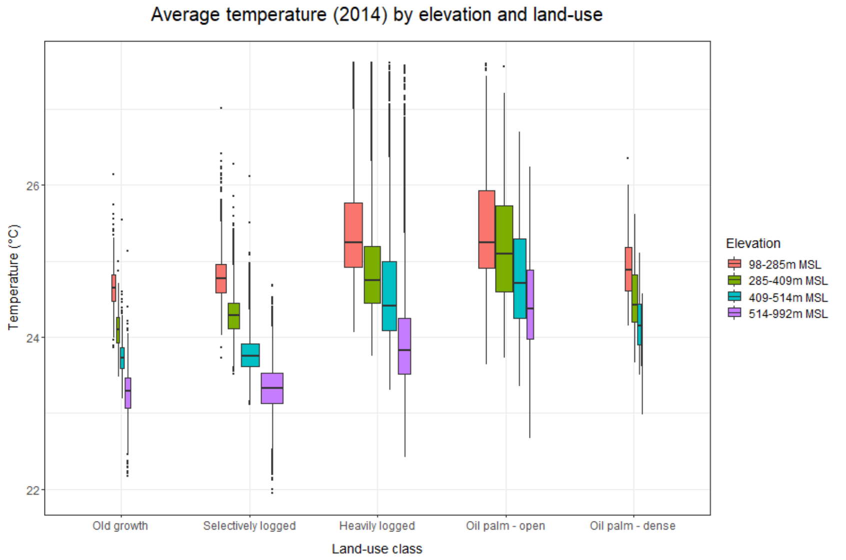


a)

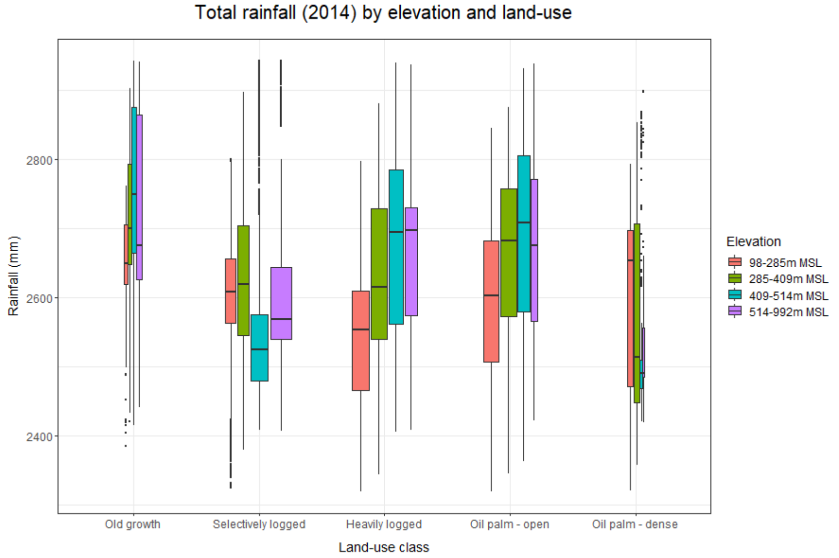


b)

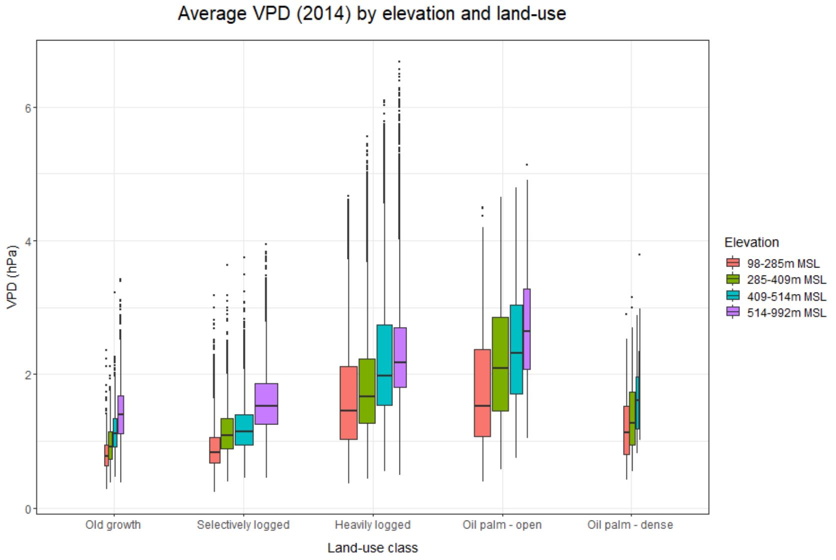


c)

Supplementary Figure S3: *annual a) mean temperature b) total rainfall and c) mean VPD stratified by land-use and elevation class. Width of the bars represents number of observations in that class.*

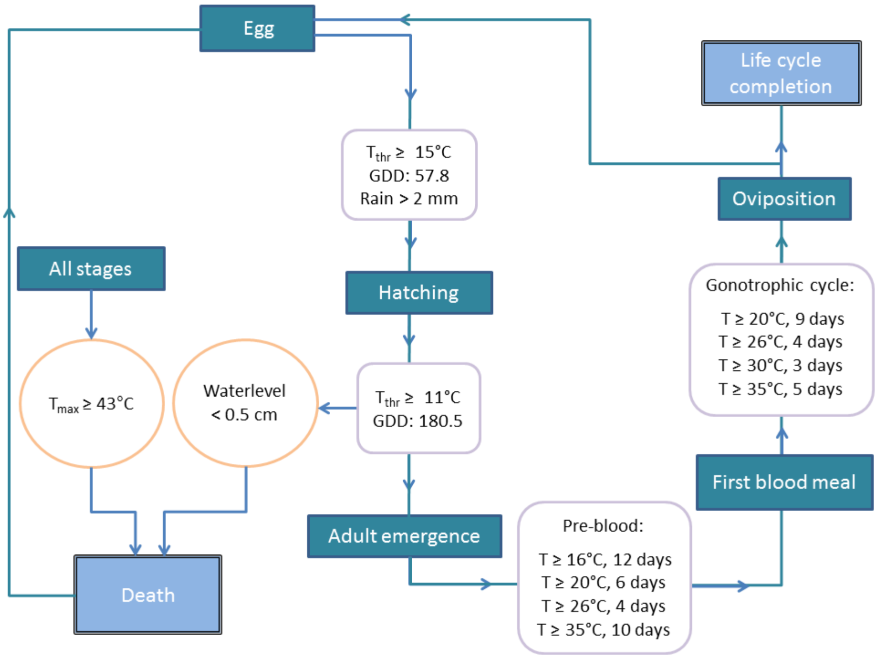


Supplementary Figure S4: *A schematic overview of our model flow. Aedes albopictus progresses from egg to immature to adult to first blood meal and then completes its life-cycle with the oviposition of a new batch of eggs. Development rates depend on the daily mean temperature. During all life-stages, mosquitoes are killed if the maximum temperature on that day exceeds our heat kill threshold. In addition, breeding sites should contain sufficient water for survival of immature life-stages (E model) and a daily precipitation threshold is set to stimulate hatching of eggs (R model). All elements are included in our combined, final model (ER model).*

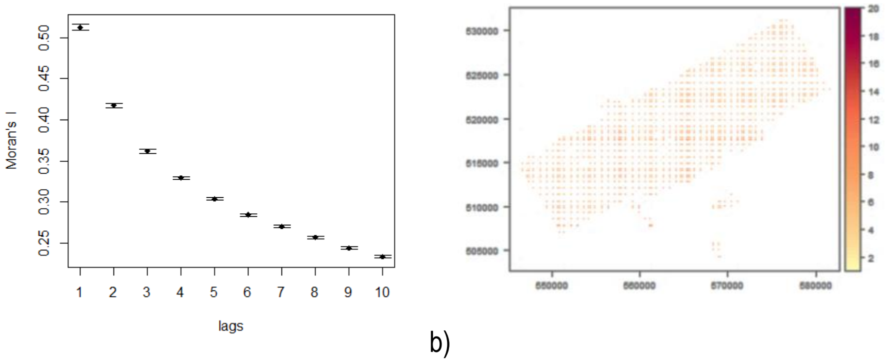


a) b)

Supplementary Figure S5: a*) Correlogram of the spatial autocorrelation in the model residuals at increasing lag distances, whereby 1 lag is 1 grid cell. b) mapping of LCC/year after sampling grid cells at 5 lag distance to reduce spatial autocorrelation.*

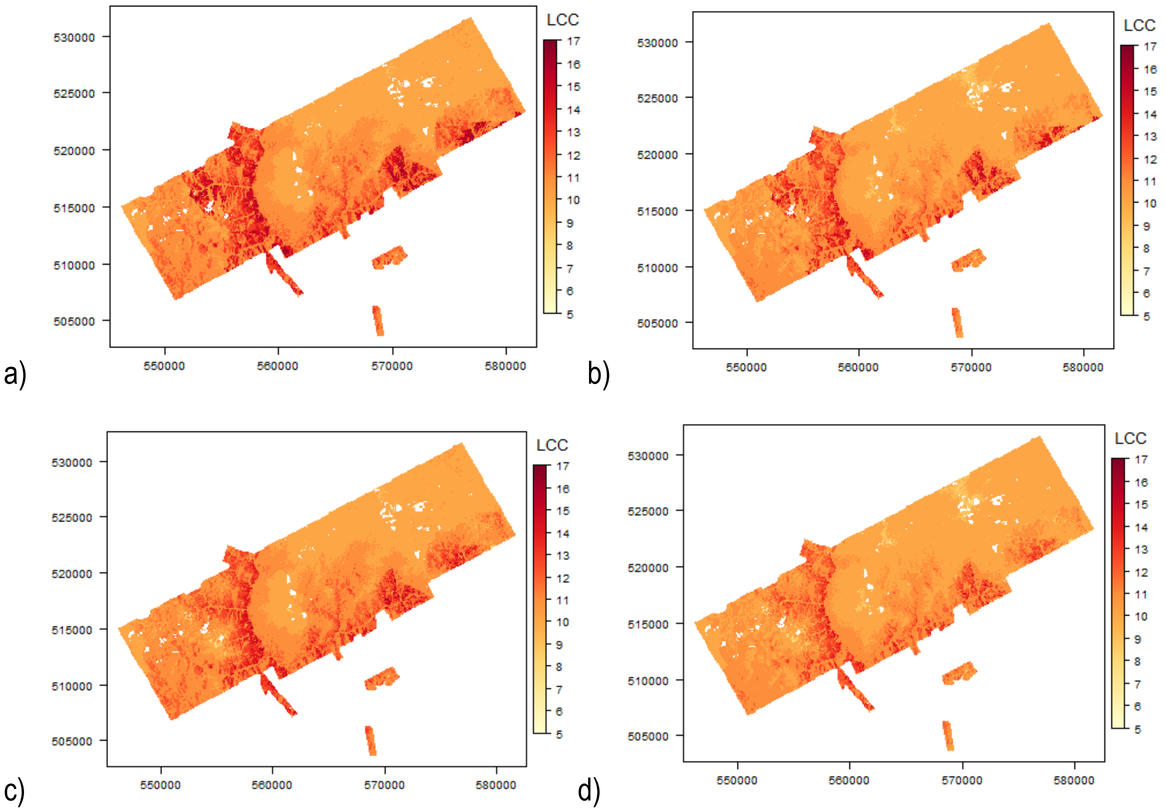


Supplementary Figure S6: *Mapping of the distribution of life-cycle completions (LCC) by A. albopictus in 2014 in the SAFE project area, Malaysian Borneo for model variations a) Base, including only temperature input b) R, including input of temperature and rainfall for egg-hatching c) E, including input of temperature and evaporation rates d) ER, combining input from temperature, rainfall and evaporation-rates.*

Table S1: *Model output for our four different model variations and sensitivity analyses for the model parameters in the combined (ER) model. The table displays mean predicted LCC across the entire study area for the year 2014 at 3 different levels of elevation. In addition, the relative (%) difference in mean LCC/year between young oil palm plantation (OY), primary forest (PF), old oil palm plantation (OO) and (for non-lagged) bare land (BL) is provided.*

*Base=base model variation, temperature-based only; E=base model + threshold for the minimum water depth in breeding sites calculated by balancing rainfall and evaporation; R=base model + daily precipitation threshold for egg-hatching; ER=combined model with all restrictions; no interaction= marginal effects without interaction terms between land-use and topography; non-lagged=model output without spatial correction; 500m=dataset aggregated to 500 instead of 50 meter resolution; D-low/high=minimum water depth for immature survival; egg-low/high=GDD for embryonation up to egg hatching; heatkill= daily maximum temperature threshold for survival; immature-low/high=GDD for immature (=larval/pupal) development up to adult emergence; R-low/high=rainfall threshold for egg hatching; W-low/high=average wind speed in the study area, determines evaporation; n.s.=non-significant: overlapping 95% confidence intervals*

| Model | Mean predicted LCC for OY//OO//PF//BL or HL | | | | | | | | | | | |
|---|---|---|---|---|---|---|---|---|---|---|---|---|
| Elevation m) | 250 | | | | 350 | | | | 450 | | | |
| **BASE** | 12.1 | 11.3 | 10.8 | HL:12.1 | 11.7 | 11.0 | 10.3 | HL:11.6 | 11.3 | 10.6 | 10.3 | HL:11.2 |
| **E** | 12.0 | 11.3 | 10.7 | | 11.5 | 11.0 | 10.3 | | 11.0 | 10.6 | 10.3 | |
| **R** | 11.6 | 10.9 | 10.4 | | 11.2 | 10.5 | 10.2 | | 10.8 | 10.1 | 10.1 | |
| **ER** | 11.5 | 10.9 | 10.4 | HL:11.2 | 11.0 | 10.5 | 10.1 | HL:10.9 | 10.6 | 10.1 | 10.1 | HL:10.5 |
| **ER no interactions** | 11.4 | 10.8 | 10.7 | | 11.1 | 10.5 | 10.4 | | 10.7 | 10.2 | 10.1 | |
| **Base non-lagged** | 12.1 | 11.3 | 10.8 | BL:13.9 | 11.7 | 10.9 | 10.5 | BL:13.3 | 11.3 | 10.6 | 190.3 | BL:12.8 |
| ER non-lagged | 11.5 | 10.8 | 10.4 | BL:12.1 | 11.0 | 10.5 | 10.2 | BL:11.4 | 10.6 | 10.1 | 10.1 | BL:10.7 |
| E non-lagged | 12.0 | 11.3 | 10.8 | BL:13.0 | 11.5 | 10.9 | 10.5 | BL:11.9 | 11.0 | 10.6 | 10.3 | BL:10.9 |
| ER 500m | 11.3 | 11.0 | 10.2 | | 10.8 | 10.1 | 10.1 | | 10.39 | 9.3 | 9.9 | |
| ER D-high | 11.5 | 10.9 | 10.2 | | 11.0 | 10.4 | 10.2 | | 10.5 | 10.0 | 10.1 | |
| ER D-low | 11.5 | 10.9 | 10.3 | | 11.0 | 10.4 | 10.2 | | 10.6 | 10.0 | 10.1 | |
| ER egg-high | 11.1 | 10.6 | 10.2 | | 10.7 | 10.3 | 10.0 | | 10.3 | 10.0 | 9.8 | |
| ER egg- low | 11.7 | 11.1 | 10.7 | | 11.3 | 10.7 | 10.5 | | 10.9 | 10.3 | 10.4 | |
| ER heatkill | 11.5 | 10.9 | 10.3 | | 11.0 | 10.4 | 10.2 | | 10.6 | 10.0 | 10.1 | |
| ER immature-high | 11.2 | 10.6 | 10.1 | | 10.8 | 10.3 | 10.0 | | 10.4 | 10.0 | 9.9 | |
| ER immature-low | 11.7 | 11.1 | 10.6 | | 11.3 | 10.7 | 10.5 | | 10.9 | 10.4 | 10.2 | |
| ER R-high | 11.1 | 10.5 | 10.1 | | 10.6 | 9.9 | 9.9 | | 10.2 | 9.3 | 9.7 | |
| ER R-low | 11.5 | 10.9 | 10.3 | | 11.0 | 10.5 | 10.2 | | 10.6 | 10.1 | 10.1 | |
| ER W-high | 9.9 | 10.2 | 10.3 | | 9.2 | 9.5 | 10.0 | | 8.5 | 8.8 | 9.7 | |
| ER W-low | 11.5 | 10.9 | 10.3 | | 11.1 | 10.4 | 10.2 | | 10.8 | 10.0 | 10.1 | |

| Model | Difference OY-PF (%) | | | Difference OO-PF (%) | | | Difference OO-OY (%) | | |
|---|---|---|---|---|---|---|---|---|---|
| Elevation (m) | 250 | 350 | 450 | 250 | 350 | 450 | 250 | 350 | 450 |
| **BASE** | 12.4 | 11.1 | 9.7 | 4.8 | 3.9 | 3.0 (n.s.) | -6.8 | -6.5 | -6.1 |
| **E** | 12.4 | 9.7 | 6.9 | 5.9 | 4.6 | 3.3 | -5.9 | -4.6 | -3.3 |
| **R** | 11.5 | 9.2 | 7.0 | 4.7 | 2.3 (n.s.) | 0.01 (n.s.) | -6.1 | -6.3 | -6.6 |
| **ER** | 10.8 | 7.9 | 5.1 | 4.7 | 2.3 | 0.01 (n.s.) | -5.5 | -5.1 | -4.8 |
| ER no interactions | 6.0 | 6.0 | 6.0 | 0.6 (n.s.) | 0.6 (n.s.) | 0.6 (n.s.) | -5.1 | -5.1 | -5.1 |
| Base non-lagged | *BL-PF:* 28.5 | *BL-PF:* 26.3 | *BL-PF:* 24.1 | | | | | | |
| ER non-lagged | *BL-PF:* 16.8 | *BL-PF:* 11.2 | *BL-PF:* 5.9 | | | | | | |
| E non-lagged | *BL-PF:* 20.2 | *BL-PF:* 13.2 | *BL-PF:* 6.6 | | | | | | |
| ER 500m | 11.1 | 7.6 | 4.2 | 8.3 | 0.7 (n.s.) | -6.5 (n.s.) | -2.5 (n.s.) | -6.5 | -10.3 |
| ER D-high | 11.4 | 7.9 | 4.6 | 5.9 | 2.5 | -0.7 (n.s.) | -5.0 | -5.0 | -5.0 |
| ER D-low | 11.5 | 8.3 | 5.1 | 5.9 | 2.5 | -0.7 (n.s.) | -5.1 | -5.3 | -5.5 |
| ER egg-high | 9.3 | 7.0 | 4.7 | 4.0 | 2.8 | 1.6 (n.s.) | -4.9 | -3.9 | -3.0 |
| ER egg- low | 9.0 | 7.3 | 5.6 | 3.0 | 1.0 (n.s.) | -0.9 (n.s.) | -5.5 | -5.9 | -6.2 |
| ER heatkill | 11.5 | 8.1 | 4.8 | 5.9 | 2.5 | -0.7 (n.s.) | -5.1 | -5.2 | -5.3 |
| ER immature-high | 10.6 | 7.6 | 4.7 | 4.9 | 2.8 | 0.8 (n.s.) | -5.1 | -4.5 | -3.8 |
| ER immature-low | 9.9 | 8.2 | 6.5 | 4.0 | 2.4 | 0.8 (n.s.) | -5.4 | -5.3 | -5.3 |
| ER R-high | 9.1 | 7.1 | 5.1 | 3.4 | -0.2 (n.s.) | -3.7 | -5.2 | -6.8 | -8.3 |
| ER R-low | 11.3 | 8.1 | 4.9 | 5.5 | 2.6 | -0.3 (n.s.) | -5.2 | -5.1 | -4.9 |
| ER W-high | -3.8 | -8.1 | -12.2 | -1.1 (n.s.) | -5.1 | -8.9 | 2.8 (n.s.) | 3.2 | 3.7 (n.s.) |
| ER W-low | 12.1 | 9.5 | 7.1 | 5.9 | 2.5 | -0.7 (n.s.) | -5.5 | -6.4 | -7.3 |
